# Supplementary material for: Wolbachia-induced Cytoplasmic Incompatibility drives epigenetic and maternally-influenced post-embryonic defects
Source: PLoS Pathog. 2026 May 7;22(5):e1014180. doi: 10.1371/journal.ppat.1014180 (PMC13152123; doi:10.1371/journal.ppat.1014180)
Supplement: S1 Table — (DOCX) [file ppat.1014180.s001.docx]

Supplemental Table 1. Sample sizes of eggs and larvae data corresponding to figure 5 C,D, and F.

| Figure 5C |  | |  |  | |
| --- | --- | --- | --- | --- | --- |
|  | Control | |  | CI | |
|  | Eggs | Larvae |  | Eggs | Larvae |
| DGRP-93 | 545 | 494 |  | 600 | 483 |
| DGRP-712 | 675 | 603 |  | 665 | 528 |
| DGRP-352 | 575 | 522 |  | 600 | 483 |
| DGRP-350 | 620 | 554 |  | 590 | 465 |
| DGRP-324 | 560 | 493 |  | 620 | 454 |
| DGRP-32 | 450 | 402 |  | 450 | 361 |
| DGRP-313 | 600 | 534 |  | 585 | 457 |
| DGRP-28 | 550 | 402 |  | 550 | 371 |
| DGRP-26 | 555 | 513 |  | 555 | 501 |
| DGRP-21 | 600 | 552 |  | 550 | 420 |
| DGRP-189 | 545 | 496 |  | 600 | 496 |
| DGRP-136 | 600 | 582 |  | 600 | 544 |
| DGRP-100 | 505 | 464 |  | 440 | 395 |
|  |  |  |  |  |  |
| Figure 5D |  |  |  |  |  |
|  | Control | |  | CI | |
|  | Eggs | Larvae |  | Eggs | Larvae |
| DGRP-93 | 765 | 691 |  | 905 | 724 |
| DGRP-352 | 1025 | 995 |  | 1075 | 829 |
| DGRP-32 | 860 | 807 |  | 820 | 596 |
| DGRP-100 | 320 | 298 |  | 275 | 210 |
|  |  |  |  |  |  |
| Figure 5F |  |  |  |  |  |
|  | Control | |  | CI | |
|  | Eggs | Larvae |  | Eggs | Larvae |
| DGRP-93 | 765 | 691 |  | 905 | 724 |
| DGRP-712 | 425 | 404 |  | 400 | 311 |
| DGRP-352 | 1025 | 995 |  | 1075 | 829 |
| DGRP-350 | 205 | 182 |  | 200 | 155 |
| DGRP-324 | 210 | 182 |  | 200 | 149 |
| DGRP-32 | 860 | 807 |  | 820 | 596 |
| DGRP-313 | 200 | 178 |  | 185 | 146 |
| DGRP-28 | 150 | 122 |  | 150 | 106 |
| DGRP-26 | 155 | 139 |  | 155 | 141 |
| DGRP-21 | 375 | 362 |  | 325 | 269 |
| DGRP-189 | 305 | 290 |  | 350 | 297 |
| DGRP-136 | 200 | 195 |  | 200 | 175 |
| DGRP-100 | 320 | 298 |  | 275 | 210 |
